# Arginine Vasotocin Regulation of Interspecific Cooperative Behaviour in a Cleaner Fish

**DOI:** 10.1371/journal.pone.0039583

**Published:** 2012-07-03

**Authors:** Marta C. Soares, Redouan Bshary, Rute Mendonça, Alexandra S. Grutter, Rui F. Oliveira

**Affiliations:** 1 Unidade de Investigação em Eco-Etologia, ISPA – Instituto Universitário, Lisboa, Portugal; 2 Université de Neuchâtel, Institut de Zoologie, Neuchâtel, Switzerland; 3 The University of Queensland, School of Biological Sciences, St. Lucia, Australia; 4 Champalimaud Neuroscience Programme, Instituto Gulbenkian de Ciência, Oeiras, Portugal; University of São Paulo, Brazil

## Abstract

In an interspecific cooperative context, individuals must be prepared to tolerate close interactive proximity to other species but also need to be able to respond to relevant social stimuli in the most appropriate manner. The neuropeptides vasopressin and oxytocin and their non-mammalian homologues have been implicated in the evolution of sociality and in the regulation of social behaviour across vertebrates. However, little is known about the underlying physiological mechanisms of interspecific cooperative interactions. In interspecific cleaning mutualisms, interactions functionally resemble most intraspecific social interactions. Here we provide the first empirical evidence that arginine vasotocin (AVT), a non-mammalian homologue of arginine vasopressin (AVP), plays a critical role as moderator of interspecific behaviour in the best studied and ubiquitous marine cleaning mutualism involving the Indo-Pacific bluestreak cleaner wrasse *Labroides dimidiatus*. Exogenous administration of AVT caused a substantial decrease of most interspecific cleaning activities, without similarly affecting the expression of conspecific directed behaviour, which suggests a differential effect of AVT on cleaning behaviour and not a general effect on social behaviour. Furthermore, the AVP-V1a receptor antagonist (manning compound) induced a higher likelihood for cleaners to engage in cleaning interactions and also to increase their levels of dishonesty towards clients. The present findings extend the knowledge of neuropeptide effects on social interactions beyond the study of their influence on conspecific social behaviour. Our evidence demonstrates that AVT pathways might play a pivotal role in the regulation of interspecific cooperative behaviour and conspecific social behaviour among stabilized pairs of cleaner fish. Moreover, our results suggest that the role of AVT as a neurochemical regulator of social behaviour may have been co-opted in the evolution of cooperative behaviour in an interspecific context, a hypothesis that is amenable to further testing on the potential direct central mechanism involved.

## Introduction

Cleaning behaviour has long been seen as a textbook example of mutualistic cooperation [1], [2]. Cleaning mutualisms are common interspecific relationships in which terrestrial vertebrates, fishes or even invertebrates act as cleaners to other individuals (so-called client species) that may include other fishes, turtles, marine iguanas and even whales [3]–[5]. In one of these mutualisms, involving the Indo-Pacific bluestreak cleaner wrasse *Labroides dimidiatus,* interactions are very frequent. *L. dimidiatus* inspect an average 2297 fish clients per day [6], a value that clearly extends beyond the number of interactions they have with conspecifics (M.S.C., R.B., A.S.G., pers. obs.). Moreover, there can be a high number of repeated cleaning interactions between the same individuals [1]. Both the high frequency and repetitive nature of these interactions should impose a selective pressure leading to the evolution of interspecific social behaviours.

Furthermore, some obligatory cleaners exhibit a set of behavioural patterns usually not observed between animals of different species that include not only the tendency to approach and/or tolerate the close proximity of interspecific individuals but also to respond to relevant interspecific stimuli in the most appropriate manner. For example, *L dimidiatus* is able to: (i) distinguish between predator and non-predator clients and between familiar and unfamiliar individuals within the same client species and to adjust cleaning behaviour accordingly [7], [8]; (ii) provide tactile stimulation to clients as a way to manipulate their behaviour therefore attracting them to, or retaining them in its cleaning territory [9]–[11]; (iii) adjust its cleaning behaviour depending on the presence or absence of third parties, becoming more cooperative if bystander clients are present [12], [13]; and (iv) feed against its preference and thus reduce an immediate reward in order to gain future benefits (i.e. temporal discounting [14]).

In contrast to the increasing knowledge on the functional aspects of cleaning mutualisms in the last decades, their underlying physiological mechanisms are virtually unknown. In one of the few studies available, Lenke [15] tested the hormonal control of cleaning behaviour. By assessing the effects of prolactin and melatonin on cleaning motivation, he showed that cleaning was partly independent from feeding motivation (i.e. hunger). Therefore, cleaning interactions should be considered not only as a particular case of feeding behaviour by cleaners but as social interactions between individuals of different species. It is thus possible that during the evolution of obligatory cleaning, which likely could have involved increasingly elaborate interactions between cleaners and their clients, the physiological mechanisms already in place for the regulation of social behaviour could have been recruited and its action extended to the regulation of interspecific interactions.

One major class of neuromodulators that is involved in the control of social behaviour that may have been co-opted for the regulation of cleaning behaviour is a group of nonapeptides of the vasopressin/oxytocin family. Arginine vasopressin (AVP) and oxytocin (OT) found in mammals and their non-mammalian homologues, arginine vasotocin (AVT), mesotocin (MT, in birds, reptiles, amphibians) and isotocin (IT, in teleost fish) [16], are presently acknowledged to play a key role as modulators of social behaviour [17], [18]. These neuropeptides are implicated in a wide range of social behaviours from aggression and reproductive behaviour, to affiliation such as maternal care, pair bonding, and social recognition [17]–[21]. The contributions of these neuropeptides to each of these behaviours may vary tremendously across species, sexes, phenotypes and social environments (reviewed by [17]). The central behavioural actions of AVT/AVP are mainly mediated by its V1a receptor subtype in both mammals and non-mammals [22]–[24]. Indeed, V1a antagonists, such as manning compound, have been observed to produce opposite effects to exogenous administrations of AVT on social behaviours [25]–[27].

In teleost fish, most empirical studies concerning the effects of neuropeptides on social behaviour have been done in the context of reproduction. For instance, AVT has been found to be positively associated with changes in courtship behaviour [27], [28], aggressive behaviour [29], [30] and in pair formation in monogamous fish [31] while specifically suppressing social behaviour in other species [17], [27], [32]. In an attempt to measure the effects of peptides on behaviour unrelated to reproduction, Thompson and Walton [33] found that exogenous administration of AVT inhibited, whereas isotocin promoted, approach behaviour towards conspecific stimuli in goldfish (*Carassius auratus*). More recently, Dewan and colleagues [34] used a comparative approach to establish associations between social behaviour and the AVT system among congeneric and shoaling butterflyfishes, more specifically the density of AVT varicosities in regions homologous to those that in mammals are known to predict social functions [35].

Here, we studied the influence of neuropeptide exogenous administration in female cleaner wrasse *L. dimidiatus* on their cleaning behaviour. These cleaners are protogynous hermaphrodites; individuals first breed as females and eventually change sex to become male harem owners [36], [37]. Males will often be found living and cleaning in pairs, usually with the largest female in its harem [36], but will frequently visit and interact with all remaining females. Cleaner fish inspect the surface, gills and sometimes the mouth of so called ‘client’ reef fish, eating ectoparasites, mucus, scales and dead or damaged tissue (reviewed by [5], [38]). Individual clients often visit cleaners several times during the same period; for example, in the rabbit fish *Siganus doliatus* this occurs on average 144 times a day [39]. When interacting with clients, cleaners are faced with the decision to cooperate by removing parasites or to otherwise cheat by eating client mucus, which they prefer [15], [40]. Consequently, conflicts arise often due to cleaners’ cheating behaviour, which can be measured by a client’s reaction, commonly referred to as jolting behaviour, in reaction to a cleaner fish’s bite [41], [42]. In these situations, cleaners may then choose to invest further in the interaction by providing tactile stimulation to clients, during which they typically massage a client’s dorsal area with their pelvic and pectoral fins [10]. These cleaners make use of a highly diverse behavioural repertoire to persuade their clientele to visit, to increase the duration of inspection and to promote a client’s return in the near future [43].

The perception and behavioural output of these cleaners will generally change in the course of each interaction with a client, which entails different demands: a) motivation to interact - whether to approach or not a client; b) investment and reward - while inspecting a client, it must decide on how much it wants to invest, whether to be honest, or if it rather prefers to aim for an easy reward (e.g. to cheat) and c) investment reinforcement - whether a cleaner is willing to invest further in the partnership whenever a client decides to leave. Neuropeptides may influence cleaners’ decision-making process along these many steps of their interactive demands. To test whether a cleaner fish’s behaviour is directly influenced by neuropeptides (i.e. AVT, isotocin and the V1a antagonist manning compound) we used an integrated approach of field manipulations and observations with a laboratorial experiment to achieve three goals. First, we aimed to determine whether exogenous neuropeptide administration (via peripheral injection) would affect a cleaner fish’s likelihood to engage in cleaning behaviour in field conditions (i.e. proportion of cleaning interactions initiated by a cleaner, proportion of clients that were inspected and proportion of cleaners switching from a current client to a newly arrived client) and whether neuropeptides directly influence cleaning service quality - a measure of degree of cooperativeness (i.e. duration that cleaners spent inspecting clients, frequency of client jolts in response to cleaner fish bites and proportion of interactions in which the cleaner fish chose to apply tactile stimulation to client). Second, in order to check that these effects are specific to the cleaning domain, we tested the effect of the same neuropeptides on social interactions with conspecific partners under natural conditions. Since the two types of interactions (interspecific and intraspecific) depend not only on the focal individual’s decisions but also on the decisions of the conspecific cleaner fish partner or the client partner, our third goal was to test in controlled laboratory conditions whether neuropeptides affected a cleaner fish’s approach response towards a conspecific vs an interspecific partner.

## Results

### a) Neuropeptide Effect on the Likelihood to Engage in Cleaning Behaviour and Cleaning Quality Levels in the Field

**Figure 1:**
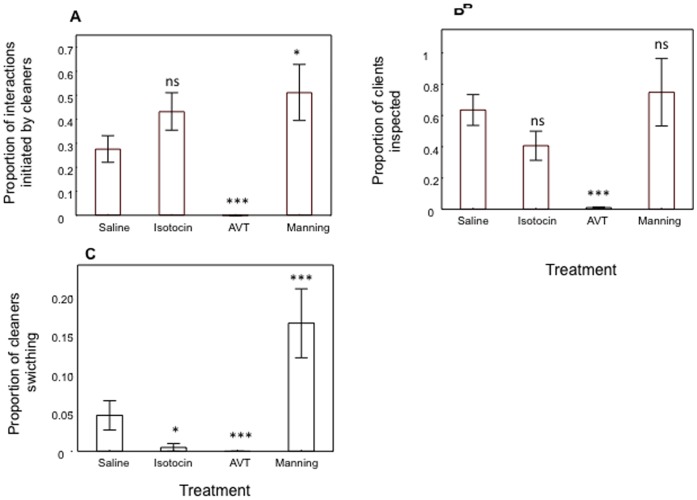
The effect of the neuropeptides isotocin, arginine vasotocin (AVT), and manning compound (Manning) on the cleaning behaviour of the cleaner fish *Labroides dimidiatus*, measured in the field and compared with a control (saline), for several behaviours. A) proportion of interactions initiated by cleaners (number of cleaning events initiated by cleaners/total number of cleaning events, B) proportion of clients that were inspected (number of clients’ cleaned/total number of visits) and C) proportion of cleaners switching from a current client to a newly arrived client (number of times cleaner switched between clients/total number of cleaning events). Measures A and C were arcsine-square root transformed to achieve normality but are presented untransformed here to facilitate visual comparisons between variables. Means are shown ±1 SEM. Symbols above bars represent P values which refer to planned comparisons of least squares means effect of each neuropeptide treatment group against the reference (saline) group (*, <0.05; ***, <0.001; ns, >0.05). The sample size for saline, isotocin and AVT was n = 8 per group, and for manning compound it was n = 6.

In field observations of interspecific cleaning behaviour, all data collected were independent measures, thus these were analysed using a one-way ANOVA (analysis of variance) with neuropeptide group as a fixed factor, followed by planned comparisons of least squares means (see Methods section). Overall, there were significant effects of treatment on all three measures of a cleaner fish’s likelihood to interact with clients: the proportion of cleaning interactions initiated by a cleaner fish (one-way ANOVA: *F*_3,26_ = 21.65, *p*<0.001, Figure 1-A), the proportion of clients that were inspected (*F*_3,26_ = 8.43, *p*<0.001, Figure 1-B), and the proportion of cleaners switching from a current client to a newly arrived client (*F*_3,26_ = 13.70, *p*<0.001, Figure 1-C).

AVT significantly decreased, whereas manning compound significantly increased the proportion of cleaning interactions initiated by cleaners (planned comparisons: AVT vs saline, *F*_1,26_ = 25.97, *p*<0.001; manning compound vs saline, *F*_1,26_ = 5.62, *p* = 0.03, Figure 1-A). No effect was found with isotocin for this variable (isotocin vs saline, *F*_1,26_ = 2.75, *p*>0.05, Figure 1-A). Moreover, AVT significantly decreased a cleaner fish’s proportion of clients inspected while no effect was found with isotocin or manning compound (AVT vs saline, *F*_1,26_ = 16.75, *p*≤0.001; isotocin vs saline, *F*_1,26_ = 2.24, *p*>0.05; manning compound vs saline, *F*_1,26_ = 0.47, *p*>0.05, Figure 1-B). Finally manning compound significantly increased the cleaners’ probability to switch between clients (manning compound vs saline, F_1,26_ = 9.66, p = 0.005) while cleaners treated with AVT and isotocin switched less between clients (AVT vs saline, *F*_1,26_ = 8.21, *p* = 0.008, isotocin vs saline, *F*_1,26_ = 6.03, *p* = 0.02, Figure 1-C).

**Figure 2:**
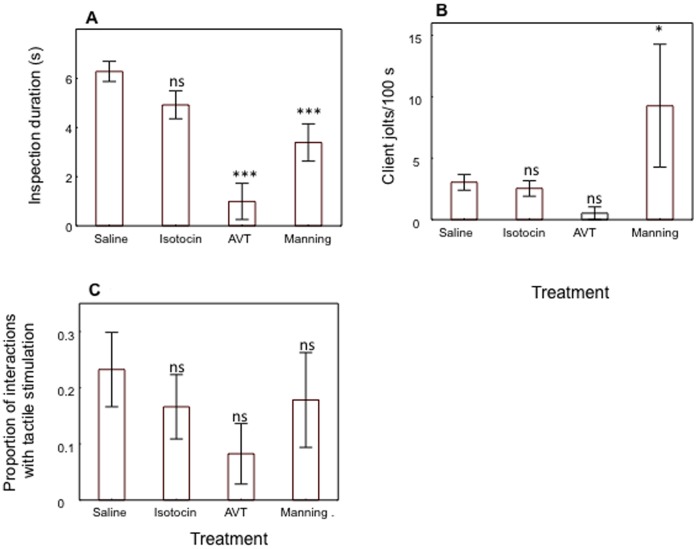
Field neuropeptide effects on measures of cleaner fish *Labroides dimidiatus* interspecific cleaning service quality. A) client inspection duration at cleaning stations (in seconds), B) number of client jolts per 100 s of inspection and C) proportion of interactions in which tactile stimulation was applied to clients (number of cleaning events in which cleaner performed tactile stimulation/total number of cleaning events). Means are shown ±1 SEM. Symbols above bars and sample sizes per treatment were the same as in Figure 1.

There were also significant effects of neuropeptide treatment on two measures of cleaning service quality: the duration of inspection (*F*_3,26_ = 14.30, *p*<0.001, Figure 2-A) and the frequency of client jolts in response to cleaner fish bites (*F*_3,26_ = 2.99, *p* = 0.049, Figure 2-B), the latter being a measure of cleaner fish cheating. No effect was found on the proportion of interactions in which the cleaner fish chose to apply tactile stimulation (*F*_3,26_ = 0.99, *p*>0.05, Figure 2-C). Both AVT and manning compound, but not isotocin, caused a significant decrease in a cleaner fish’s inspection duration when compared to the saline treatment (AVT vs saline, *F*_1,26_ = 39.07, *p*<0.001; isotocin vs saline, *F*_1,26_ = 2.59, *p*>0.05; manning compound vs saline, *F*_1,26_ = 10.01, *p* = 0.003, Figure 2-A). Only manning compound significantly increased clients’ body jolts when compared with saline treatment (AVT vs saline, *F*_1,26_ = 0.81, *p*>0.05; isotocin vs saline, *F*_1,26_ = 0.03, *p*>0.05; manning compound vs saline, *F*_1,26_ = 4.27, *p* = 0.048, Figure 2-B).

### b) Neuropeptide Effect on a Cleaner Fish’s Likelihood to Interact with Conspecific Partners in the Field

**Figure 3:**
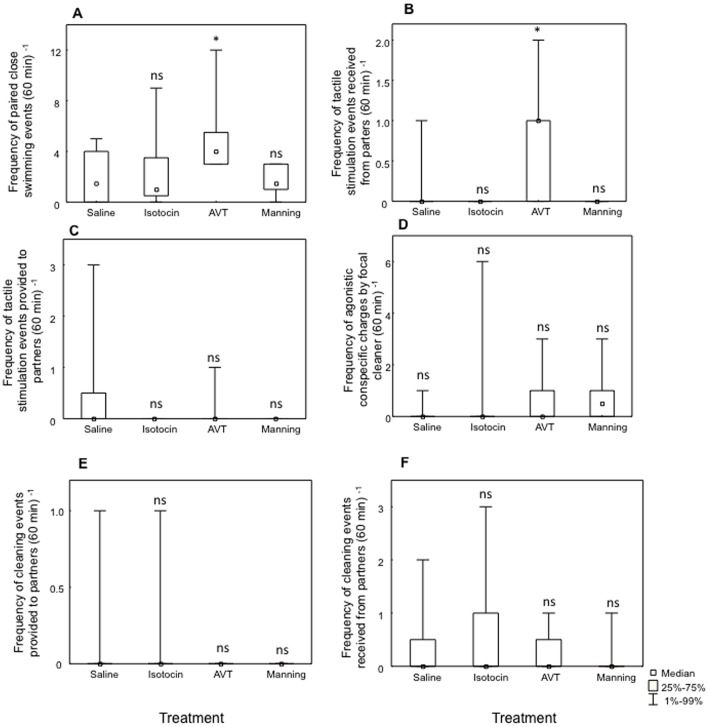
Field neuropeptide effects on measures of cleaner fish *Labroides dimidiatus* conspecific related behaviour (all per 60 minute observation). A) frequency of paired-close swimming events, B) frequency of tactile stimulation events received from partners, C) frequency of tactile stimulation events provided to partners, D) frequency of agonistic conspecific charges by focal cleaner, E) frequency of cleaning events provided to partners and F) frequency of cleaning events received from partners. Medians and interquartile ranges are shown. Symbols above bars and sample sizes per treatment were the same as in Figure 1.

Field observations of cleaner fish behaviour directed at conspecifics were independent measures and were analysed using a one-way Kruskal-Wallis analysis of variance, followed by planned Mann-Whitney *U* tests (see Methods). Overall, there was a significant effect of treatment on the frequency in which focal cleaners were observed to swim closer together (paired) with their conspecific partners (Kruskal-Wallis test: χ2 = 8.23, df = 3, *p = *0.04, Figure 3-A) and on how frequently these received tactile stimulation from their conspecific partners (χ2 = 9.12, df = 3, *p* = 0.02, Figure 3-B). In contrast, no effect of treatment was found on the remaining behavioural measures for conspecific interactions (i.e. frequency of tactile stimulation events provided to partners: χ2 = 3.57, df = 3, *p*>0.05, Figure 3-C; frequency of agonistic conspecific charges by focal cleaner: χ2 = 3.22, df = 3, *p*>0.05, Figure 3D; frequency of cleaning events provided to partners: χ2 = 1.81, df = 3, *p*>0.05, Figure 3-E; frequency of cleaning events received from partners: χ2 = 0.36, df = 3, *p*>0.05, Figure 3-F). AVT injected cleaners were more often seen swimming in close contact with their partners when compared to those injected with saline (Mann–Whitney test: AVT vs saline, *U = *12.5, *p = *0.04; Figure 3-A) whereas no effects were found with isotocin or with manning compound (isotocin vs saline, *U* = 31.0, *p*>0.05; manning compound, *U* = 24.0, *p*>0.05; Figure 3-A). Focal cleaner fish injected with AVT also received more tactile stimulation from their conspecific partners than those injected with saline (AVT vs saline, *U* = 15.5, *p* = 0.04; Figure 3-B) and again no effects were found for isotocin or for manning compound (isotocin vs saline, *U* = 28.0, *p*>0.05; manning compound, *U* = 21.0, *p*>0.05; Figure 3-B).

### c) Neuropeptide Effect on a Cleaner Fish’s Social Motivation in Captivity

**Figure 4:**
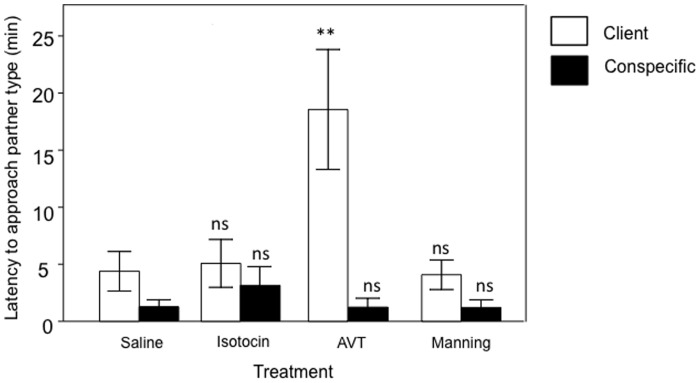
Neuropeptide effect on cleaner fish *Labroides dimidiatus* latency in time to approach partner type. Client *Zebrazoma desjardiini* (white bars) or conspecific (black bars) stimuli. Means are shown ±1 SEM. Symbols above bars represent P values which refer to planned comparisons of least squares means effect of each neuropeptide treatment group against the reference (saline) group (**, <0.01; ns, >0.05). Sample sizes were n = 8 per group.

In these captivity experiments the same cleaner fish were used for all treatment groups thus data were analysed using two-way Repeated Measures ANOVA, followed by planned comparisons. We found a significant interaction between the effects of treatment and partner type (client or conspecific) on cleaner fish’s latency in time to approach a social partner (*F*_3,42_ = 5.30, *p = *0.003, Figure 4). Only cleaners injected with AVT showed a significant increase in latency to approach client stimulus (planned comparisons: AVT vs saline, *F*_1,14_ = 10.64, *p* = 0.006; isotocin vs saline, *F*_1,14_ = 0.08, *p*>0.05; manning compound vs saline, *F*_1,14_ = 0.05, *p*>0.05; Figure 4). In contrast, none of our individual treatments produced distinctive effects on cleaners’ latency to approach the conspecific stimulus when compared to control levels (AVT vs saline, *F*_1,14_ = 0.001, *p*>0.05; isotocin vs saline, *F*_1,14_ = 0.56, *p*>0.05; manning compound vs saline, *F*_1,14_ = 0.003, *p*>0.05; Figure 4).

## Discussion

Our findings show, for the first time, a causal association between the modulatory effects of AVT and subsequent changes of a cleaner fish’s behavioural response (for a summarised view of all the results, please see Table 1). We demonstrate that AVT has effects on measures of a cleaner fish’s likelihood to interact with clients and on measures of quality of service provided (i.e. inspection time and cheating). Moreover, animals treated with the antagonist (manning compound) were more prone to interact with clients. For the measures of cleaning quality (i.e. degree of cooperativeness), manning compound mediated a rise in a cleaner fish’s motivation to cheat while it reduced the duration of time it spent with clients since such cleaner fish switched between clients more frequently (Table 1). The closely related neuropeptide isotocin had little effect on the willingness to interact with clients and none on the quality of the service (Table 1). Moreover, AVT injected cleaners were more often observed swimming in close contact with their conspecific partners and receiving more tactile stimulation (Table 1). This clearly indicates that AVT effects tend to be mostly specific to interspecific cooperative interactions rather than representing a general regulatory mechanism of social interactions irrespective of the type of partner involved (i.e. inter- vs. intra-specific). Finally, we tested in controlled conditions, a cleaner fish’s latency to react to either conspecific or interspecific stimuli as a proxy of pro-social motivation. Again, only AVT had a significant effect and increased a cleaner fish’s latency in time to approach a client partner (Table 1).

**Table 1:** Manipulations of arginine vasotocin (AVT), isotocin (IT) and manning compound injected into the cleaner fish *Labroides dimidiatus* compared with saline (control).

Behavioural response			Subjectsanalysed	AVT	IT	ManningCompound
Interspecific	Observations in the wild					
	1) Likelihood to engage incleaning behaviour					
		a) Proportion of interactions initiated by cleaners(Figure 1-A)	Cleaner	↓	↔	↑¥
		b) Proportion of clients inspected (Figure 1-B)	Cleaner/Client	↓	↔	↔
		c) Proportion of cleaner switching from a currentclient to newly arrived client (Figure 1-C)	Cleaner	↓	↓	↑¥
	2) Cleaning service quality					
		a) Inspection duration (Figure 2-A)	Cleaner/Client	↓*	↔	↓*
		c) Frequency of client jolts in response tocleaner bites/100s (Figure 2-B)	Client	↔	↔	↑
		b) Interactions in which the cleaners choose toapply tactile stimulatio to clients (Figure 2-C)	Cleaner	↔	↔	↔
	Observations in thelaboratory					
		a) Latency in time to approach a partner (Figure 4)	Cleaner	↑	↔	↔
Intraspecific	Observations in the wild					
		a) Frequency of paired close-swimming events(Figure 3-A)	Cleaner	↑	↔	↔
		b) Frequency tactile stimulation events receivedfrom partners (Figure 3-B)	Cleaner	↑	↔	↔
		c) Frequency of tactile stimulation eventsprovided to partners (Figure 3-C)	Cleaner	↔	↔	↔
		b) Frequency of agonistic conspecific charges byfocal cleaner (Figure 3-D)	Cleaner	↔	↔	↔
		e) Frequency of cleaning events provided topartners (Figure 3-E)	Cleaner	↔	↔	↔
		f) Frequency of cleaning events received frompartners (Figure 3-F)	Cleaner	↔	↔	↔
	Observations in thelaboratory					
		a) Latency in time to approach a conspecific(Figure 4)	Cleaner	↔	↔	↔

Arrows indicate the effect, relative to saline, on the behaviours of interest: ‘↑’ denotes an increase in display, ‘↓’ a decrease, and ‘↔’ indicates no effect detected).

Notes: *In this situation the reasons underlying the decrease in time spent cleaning for both AVT and manning compound treated individuals are quite different to the other situations (labelled with ¥): cleaners injected with AVT decrease their general willingness to interact and spend less time inspecting (cleaning) their visiting clientele. ¥ However, cleaners treated with the antagonist (manning compound) interacted more, switched more frequently from client to client and thus spent less time inspecting clients (see variables).

### a) Mechanisms of Arginine Vasotocin Action

To date, one isotocin and three distinct AVT receptors (i.e. V1a1, V1a2, and V2) have been characterized in teleost fish but their functional roles have not been clearly established yet [24]. Similar to what happens in mammals [44], the V1a-type receptors are also the most predominant AVT receptors expressed in the teleost brain [24], [47], and therefore are the major candidates to mediate behavioural responses in fish. In our study, both AVT and manning compound, a commonly used antagonist of the AVP type 1a receptors (V1a) that also has affinity for the oxytocin receptor in mammals [45], had a significant impact on different aspects of cleaning behaviour. However, since isotocin has little measurable effects on cleaner fish behaviour, it is acceptable to assume that the observed effects of the manning compound on behaviour were mediated by V1a-type receptors, hence confirming the neuro-behavioural role of these types of receptors.

Our results also suggest that these peptides, when administered peripherally, are able to cross the blood-brain barrier in fish. In mammals, neuropeptides are unable cross the blood-brain barrier under regular physiological conditions [46]. Although fish also have a functional blood-brain barrier that is homologous to that of mammals [47], it has different mechanisms and associated differences in permeability [48] that apparently allow the passage of systemic neuropeptides into the brain compartment. And indeed, there is a vast body of relevant literature in which AVT effects on social behaviour, social status, partner preferences, courtship, aggression and social communication have been demonstrated using peripheral administration of AVT and its antagonist manning compound [27]–[34], [49].

### b) Arginine Vasotocin Modulation of Interspecific Cleaning Behaviour

AVT effects caused a substantial decrease in a cleaner fish’s willingness to approach and inspect clients, which were in line with previous studies on the effect of AVT/AVP on pro-social behaviour [50], [51]. On the other hand, the administration of manning compound, and the putative subsequent suppression of endogenous AVT via the blocking of the V1a-type receptors, produced a clear increase in a cleaner fish’s willingness to inspect more clients and to engage in cleaning by its own initiative. A similar increase in social approach motivation influenced by manning compound has also been reported in goldfish males [34]. However, the rise in the motivation to interact in cleaner fish under the influence of the antagonist was not linked to a similar increase in the quality of service provided. On the contrary, client jolt rates (a client behavioural correlate in response to cheating by the cleaner [41], [42]) increased significantly. Clients terminate interactions in response to jolts [41], and such responses together with the increased occurrence of cleaners switching between alternative clients may explain why administration of manning compound reduced the average time spent with clients.

Overall, our results suggest a significant role for AVT as a key regulator of interspecific cleaning behaviour in this cleaner fish. The effects of AVT/AVP have been associated with social withdrawal in response to the perception of threatening stimuli [52]. For example, in humans, AVP has been noted to be responsible for an increase in the subjective perception of threat even in response to neutral stimuli [53], [54]. Thus, by affecting the motivation to interact with potential social partners, cleaner fish under the influence of higher AVT levels may perceive visiting clients as unsafe partners and as a source of a potential threat. The effects of the manning compound are in line with the hypothesis that by suppressing specific effects of endogenous AVT, via the blocking of the V1a-type receptors, cleaners increase their motivation to interact. It is perhaps this change in perception (from perceiving social partners as a threat to perceiving them as non-threatening) that creates the conditions that enable cleaners to disregard the necessity to invest in longer client inspections and to control for their dishonest tendencies. On the other hand, one potential effect of AVT/AVP is to activate the corticotrophin-releasing hormone (CRH) of the hypothalamic–pituitary–adrenal axis, leading to the release of glucocorticoid hormones, which in our fish would mean a rise of their cortisol levels [55]. Nevertheless, we believe that the increase of cortisol levels would unlikely be solely responsible for our present results, based on our recent testing involving steroid hormones in this system (cortisol and an antagonist), in which no such suppression of cleaning behaviour has been observed (M.C. Soares et al., unpublished data).

The administration of isotocin did not induce opposite effects to those seen with AVT, as predicted from earlier studies on social behaviour [20], [34]. Indeed, isotocin failed to cause a significant effect on most measures of cleaning, both in the field (in most measures of likelihood to interact and all measures of cleaning quality) and in laboratory conditions (Table 1). Generally, isotocin tended to inhibit cleaner fish’s behavioural response (which was solely significant for the measure of cleaner switching, Figure 1-C, Table 1), with the exception of a trend to increase a cleaner fish’s initiative towards interacting with clients (Figure 1-B). Interestingly, Thompson and Walton [34] also reported an isotocin effect on goldfish towards an inhibition of social approach but solely among highly social fish. Future studies should further look into inter-individual differences on the influence of isotocin in cleaner fish behaviour.

### c) Arginine Vasotocin Regulation of a Cleaner fish’s Behaviour Towards Conspecifics

In their natural habitat, cleaner fish do not solely spend time interacting with interspecific social partners, but are also part of a social system (harem-like) in which conspecific interactions are relatively frequent [37], [38]. AVT did not produce similar suppressing effects on conspecific interactions to those seen on a cleaner fish’s likelihood to engage in cleaning with their clients. On the contrary, our results show an increase in the predisposition to interact with conspecific partners (i.e. paired close swimming) that are reciprocated by a rise in the levels of tactile stimulation received (Fig. 3A, Fig. 3B, Table 1). Indeed, the role of AVP in the enhancement of social recognition has been demonstrated by the finding of the naturally occurring AVP-deficient Brattleboro rat, which displays a total disruption of social recognition [56]. Moreover, in centrally infused male and female prairie voles, AVP facilitates pair bond formation in the absence of mating [57], [49]. These effects of AVT on social recognition are thus particularly important for the establishing of partner preference mechanisms and pair bonding. In teleost fish, the effects of exogenous AVT administration on conspecific-direct behaviour have also been described. For example, AVT systemic injections in field conditions increased male courtship behaviour in both territorial and non-territorial terminal phase bluehead wrasse males [27], and increased female courtship behaviour in a sex-role reversed species, the peacock blenny [29]. In our study, AVT may be responsible for the modulation of conspecific pro-social approach in the wild and for the enhancement of pair-boding mechanisms when partnerships are already established. However, field data might not be directly comparable to those collected in laboratorial conditions: cleaner fish individuals used for laboratory experiments were not familiarized with each other, contrarily to the established pairs in the field, and therefore pair-bonding mechanisms were not in place. This hypothesis requires further field and laboratory studies specifically aimed at this goal.

### d) Concluding Remarks

Most studies concerning the effects of neuropeptides on social behaviour focus on conspecific-directed behaviour, which usually occurs in the context of reproduction (e.g. [20]). The absence of the key reproductive component may explain the lack of relevant effects produced by isotocin upon these cleaners’ behaviour. AVT revealed to have a relevant role in reducing most related interspecific cleaning activities and modulating cleaners’ dishonesty via central effects on the V1a-type receptors. However, the systemic increase of AVT did not suppress all pro-social behaviour non-specifically, as demonstrated by some of the measures related to conspecific behaviour (see Table 1). Taken together, our evidence demonstrates that AVT pathways might play a pivotal role in the regulation and promotion of interspecific cooperative behaviour and conspecific social behaviour among stabilized pairs of cleaner fish. We hypothesize that the endogenous levels of AVT should directly modulate perceptive, motivational and cognitive mechanisms that, in turn will affect cleaner fish behaviour, both in conspecific social relationships and interspecific cooperative interactions. Also, the influence of social context and the behaviour of other conspecifics and/or clients should produce changes in levels of AVT expression and release across different brain areas responsible for the regulation of multiple forms of social behavior [58]–[60]. We suggest that during the evolution of obligatory cleaning behaviour the AVT physiological mechanisms already in place for the regulation of social behavior could have been recruited and its action extended to the regulation of interspecific interactions. The following important steps are still necessary to fully understand the potential mechanisms of AVT central actions on cleaner fish behaviour: a) to compare relative actions of AVT and manning compound injections on specific brain regions (between intraspecific social behaviours and interspecific cleaning/cooperative behaviour); b) to compare these effects on other species of closely related wrasse (Labridae) species that vary in the expression of cleaning behaviour (obligatory, facultative and non-cleaners) and c) to extend this knowledge to other species of highly social teleost fishes such as some species of gobies (such as the Caribbean cleaning gobies *Elacantinus* spp) that are known to have cleaners and non-cleaners within the same species.

## Methods

### Field Methods and Behavioural Observations

Field experiments were carried out on seven different reefs around Lizard Island (Lizard Island Research Station, Australia, 14° 40'S, 145° 28′E) between August and September 2010, in which 40 female cleaner fish were tested. All manipulations and observations were made by two SCUBA divers, between 10∶00 and 16∶00 hours. Cleaner fish were selected haphazardly across the reefs and cleaning stations varied in depth between 1.5 and 12 m. Individuals were captured using a barrier net and measured to the nearest mm (TL-total length). TL of the fish ranged from 6.2 to 8.5 cm. Body weight was then estimated from a length-weight regression (unpublished data). We then gave the focal female an intramuscular injection of one of four compounds: a) saline (0.9 NaCl); b) AVT (V0130– Sigma), isotocin (H-2520 - Bachem) or Manning compound (V2255– Sigma- [β-Mercapto-β,β cyclopentamethylenepropionyl1, O-me-Tyr2, Arg8]-Vasopressin). Injection volumes ranged from 25 to 80 µl per gram of body weight (gbw). This process never exceeded 3 min. Once an individual was released it was then observed for the next 60 min. The order of the treatments was randomized for each dive and all treatments used independent cleaner fish. The dosages were based on a preliminary study done also on cleaner fish *L. dimidiatus* the year before, at Ras Mohammed National Park, Egypt where several dosages of each of our candidate neuropeptides were tested (0.5, 2.5 and 5 µg per gbw) and, all were 2.5 µg per gbw. Observations were made from a distance of 2–3 m. During each observation, we recorded the following measures: a) species and TL of each client (estimated visually to the nearest cm) visiting the cleaning station, and whether it adopted the species-specific immobile pose, which signals the need to be cleaned [61], before or after the onset of cleaning by the cleaning fish; b) the duration (in s) of a cleaner’s inspection towards each client and the number of tactile stimulations provided (where a cleaner touches, with ventral body and fins, the body of the client and no feeding is involved); c) the number of jolts by clients and the client’s reaction following each jolt; d) conspecific-directed behaviour such as: swimming closely with partner, provided or received tactile stimulation, inspected or cleaned by partner, and agonistic interactions, including charges where one individual rapidly advanced towards the other partner.

### Laboratory Experimental Methods and Behavioural Analysis

Experiments were conducted at the fish housing facilities of the Oceanário de Lisboa (Lisbon, Portugal). We used 8 wild caught *L. dimidiatus* that originated in Maldives and were directly imported to Portugal by a local distributor. The fish were kept in individual aquaria (100×40×40 cm) of a flow through system that pumped water from a larger cleaning tank (150×50×40 cm) that served as a natural filter. Nitrite concentration was kept to a minimum (always below 0.3 mg/l). Each tank contained an air supply and a commercial aquarium heater (125 W, Eheim, Jäger). Small PVC pipes (10–15 cm long; 2.5 cm diameter) served as shelter for the fish. Experiments were carried out between October 2010 and January 2011 in the individual tanks of each fish. Each aquarium was divided into two compartments separated by a removable opaque partition. Each cleaner was weighed before the onset of the experiment so that injection volume could be adjusted to body weight. The treatments and dosages used were the same as the field study (i.e. saline, AVT, isotocin, and manning compound; 2.5 µg of drug per gbw for each treatment). Treatment order was randomized. On each test day, a client (surgeonfish *Zebrassoma desjardiini*) or a conspecific *L. dimidiatus* were introduced on the other side of the experimental tank and left for at least 5 min until normal behaviour was restored. Then the focal cleaner fish would be quickly removed from its side of the tank with a hand net, injected with one of the neuropeptides tested and put back in its side of the experimental tank. This procedure never took more than 2 minutes. Injected cleaners were then left to recuperate to normal levels of swimming and opercula movements (which usually happened in under 5 min) and only then would the partition be removed and cleaners were free to approach stimuli (either a client or conspecific). All behaviour was then videotaped for the next 45 minutes while the experimenter left the room. Video recordings were analysed using the software package Noldus Observer XT (Noldus Information Technology).

### Statistical Analysis

We investigated differences between the two field observers by comparing the following measures: a) proportion of interactions initiated by cleaners, b) proportion of clients that were inspected; c) proportion of cleaner switching from a current client to newly arrived client; d) inspection duration; e) frequency of client jolts in response to cleaner bites and f) proportion of interactions in which the cleaners choose to apply tactile stimulation to clients, using a series of Independent Measures T-tests. There were no significant differences between observers in all the variables considered.

In field observations of interspecific cleaning behaviour, all cleaner fish were randomly selected and were independent measures. Interspecific cleaner fish behaviour towards clientele was measured along two different behavioural categories: a) measures of likelihood to interact with clients and b) measures of cleaning quality, a measure of degree of cooperativeness. Each of these measures in turn has several behavioural correlates. Hence, we measured cleaners’ likelihood to interact as: 1) proportion of cleaning interactions initiated by cleaners, 2) proportion of inspected clients and 3) proportion of cleaners switching from a current client to a newly arrived client. Measures of cleaning service quality included: 1) mean duration of inspection by cleaners, 2) frequency of jolts per 100 sec of inspection and 3) proportion of interactions in which tactile stimulation was used by cleaners. The proportion of interactions initiated by cleaners and proportion of cleaners switching were transformed by taking the arcsine-square root of a number to achieve a normal distribution. Data were analysed using a one-way ANOVA with neuropeptide groups as a fixed factor. ANOVA results were followed by planned comparisons of least squares means in order to compare each neuropeptide treatment with the control (saline) group.

Field observations of a cleaner fish behaviour directed at conspecifics were independent measures. However, because these behaviours were less frequent than interspecific ones, assumptions for parametric testing were not met thus non-parametric analyses were used. Data were analysed using a one-way Kruskal-Wallis analysis of variance, followed by planned Mann-Whitney *U* tests to search for specific differences between each neuropeptide manipulation and the control (saline) group.

In the laboratory experiments, the same cleaners were used in all treatment groups. Data were analysed using two-way Repeated Measures ANOVA with treatment (saline, AVT, isotocin, manning compound) and stimuli (conspecific, client) as between subject factors, followed by planned comparisons of least squares means within each factor.

All statistical tests shown in this study were two tailed.

### Ethical Commitment

Ethical clearance to work at Lizard Island Research Station (Australian Museum), involving animal experimentation, was obtained from the University of Queensland Animal Ethics Committee (Native and exotic wildlife and marine animals) – permit no SBS/104/10 (project name: “Linking behaviour and physiology in marine cleaning mutualism”). The use of animals and data collection complied with the laws of Australia, Portugal and Switzerland.
